# An Information-Theoretic Analysis of the Cost of Decentralization for Learning and Inference under Privacy Constraints

**DOI:** 10.3390/e24040485

**Published:** 2022-03-30

**Authors:** Sharu Theresa Jose, Osvaldo Simeone

**Affiliations:** Department of Engineering, King’s College London, London WC2R 2LS, UK; osvaldo.simeone@kcl.ac.uk

**Keywords:** vertical federated learning, Bayesian learning, information-theoretic analysis

## Abstract

In vertical federated learning (FL), the features of a data sample are distributed across multiple agents. As such, inter-agent collaboration can be beneficial not only during the learning phase, as is the case for standard horizontal FL, but also during the inference phase. A fundamental theoretical question in this setting is how to quantify the cost, or performance loss, of decentralization for learning and/or inference. In this paper, we study general supervised learning problems with any number of agents, and provide a novel information-theoretic quantification of the cost of decentralization in the presence of privacy constraints on inter-agent communication within a Bayesian framework. The cost of decentralization for learning and/or inference is shown to be quantified in terms of conditional mutual information terms involving features and label variables.

## 1. Introduction

**Figure 1:**
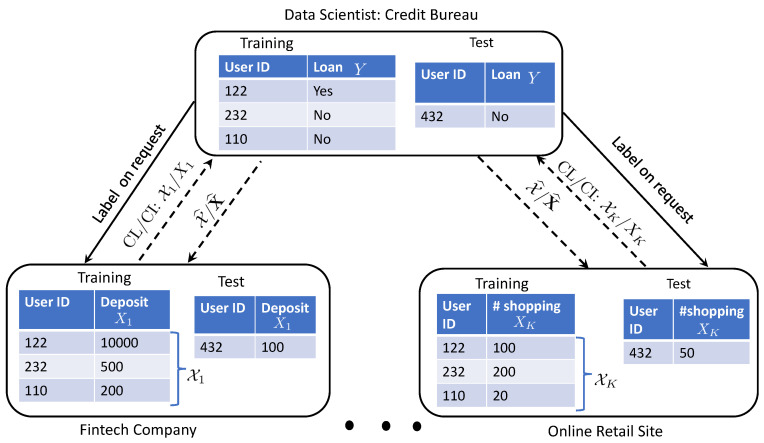
Illustration of the vertical federated learning (FL) setup under study for a prototypical credit scoring application.

Consider a digital bank interested in building a prediction model for credit scoring based on data features of given individuals, such as saving information and spending habits, that are distributed across other banks, fintech companies, and online retail shops (see Figure 1). Data labels indicating loan approval or rejection reside at a trusted third-party credit bureau, which keeps track of the approved loans [1]. This setting exemplifies vertical federated learning (FL), in which data features are scattered across different participating agents, with data barriers between them preventing a direct exchange of information.

Unlike conventional horizontal FL, in which agents have independent data points, in vertical FL settings, inter-agent collaboration can be beneficial not only during the learning phase but also during the inference phase [2,3]. It is therefore important to understand at a fundamental theoretical level whether decentralization, wherein agents use only local data for learning and/or inference, entails a significant performance loss as compared to collaborative learning and/or inference. This is the subject of this paper.

As a first attempt in this direction, Chen et al. [3] address this problem by studying a binary classification problem in which each class corresponds to a bivariate Gaussian distribution over two input features, which are vertically distributed between two agents. The authors identify four collaboration settings depending on whether collaboration is done during learning and/or inference phases as collaborative learning–collaborative inference (CL/CI), collaborative learning–decentralized inference (CL/DI), decentralized learning–collaborative inference (DL/CI), and decentralized learning–decentralized inference (DL/DI). By taking a frequentist approach, the authors compare the classification error rates achieved under these four settings.

In this work, inspired by [3], we develop a novel *information-theoretic* approach to quantify the cost of decentralization for *general* supervised learning problems with *any* number of agents and under *privacy* constraints. Specifically, we consider a supervised learning problem defined by an arbitrary joint distribution $P_{X,Y|W}$ involving the feature vector X and label *Y*, with the feature vector vertically partitioned between any number of local agents. A trusted central server, also called a data scientist or aggregator [4], holds the labels, which it shares with the agents upon request (see Figure 1). The agents collaborate through the aggregator during learning and/or inference. To limit the information leakage from the shared feature to an adversarial eavesdropper, unlike [3], privacy constraints are imposed on the aggregation mapping. By adopting a Bayesian framework, we characterize the average predictive performance of the four settings—CL/CI, CL/DI, DL/CI, and DL/DI—under privacy constraints via information-theoretic metrics. Finally, we illustrate the relation between the four collaboration settings with/without privacy constraints on two numerical examples.

In line with the recent works of [5,6], this work relates information-theoretic measures to learning centric performance metrics with the goal of providing theoretical insights. Specifically, we leverage information-theoretic tools to gain insights into the performance degradation resulting from decentralized learning and/or inference for general supervised learning problems. The main contribution is hence of theoretical nature, as it provides a connection between information-theoretic metrics and practically relevant measures of generalization in decentralized Bayesian learning and inference.

## 2. Problem Formulation

**Setting:** We study a vertical federated learning (FL) setting with *K* agents that can cooperate during the learning and/or inference phases of operation of the system. Our main goal is to quantify, using information-theoretic metrics, the benefits of cooperation for learning and/or inference. We focus on a supervised learning problem, in which each data point corresponds to a tuple (X,Y) encompassing the *K*-dimensional feature vector $X=(X_{1},\ldots ,X_{K})$ and the scalar output label *Y*. As illustrated in Figure 1, each *k*th feature $X_{k}$ in vector X is observed only by the *k*th agent. A trusted central server, referred to as the aggregator, holds the output label *Y*, which it shares with the agents on request [4,7]. Features and labels can take values in arbitrary alphabets. The unknown data distribution is assumed to belong to a model class $\left\{P_{X,Y|W}:W\in W\right\}$ of joint distributions that are identified by a model parameter vector *W* taking values in some space W. Adopting a Bayesian approach, we endow the model parameter vector with a prior distribution $P_{W}$.

As illustrated in Figure 1, let $\left(X,Y\right)=\{\left(X_{1},Y_{1}\right),\ldots ,\left(X_{N},Y_{N}\right)\}$ denote a training data set of *N* labelled samples, which, when conditioned on model parameter *W*, are assumed to be generated i.i.d. according to distribution $P_{X,Y|W}$. The N×K matrix X collects the *K*-dimensional feature vectors ${\left\{X_{n}\right\}}_{n=1}^{N}$ by rows. We denote as $X_{n,k}$, the (n,k)th element of matrix X, for n=1,…,N, and k=1,…,K; and as $X_{k}={\left[X_{1,k},\ldots ,X_{N,k}\right]}^{T}$ (${\left[\cdot \right]}^{T}$ is the transpose operation), the *k*th column of the data matrix, which corresponds to the observations of agent *k*. The goal of the system is to use the training data set (X,Y) to infer the model parameter *W*, which enable the agents to predict the label of a new, previously unseen, test feature input X. The joint distribution of model parameter *W*, training data (X,Y), and test data (X,Y) can be written as follows ([8], Chapter 3.3):

(1) $$\begin{array}{c} P_{W,X,Y,X,Y}=P_{W}{⊗}_{i=1}^{N}\underset{\mathrm{training}}{\underset{︸}{\left(P_{X_{i,1},\ldots ,X_{i,K},Y_{i}|W}\right)}}⊗\underset{\mathrm{testing}}{\underset{︸}{P_{X_{1},\ldots ,X_{K},Y|W}}}, \end{array}$$

with ⊗ representing the product of distributions, and conditional distribution $P_{X_{i,1},\ldots ,X_{i,K},Y_{i}|W}$ being equal to $P_{X_{1},\ldots ,X_{K},Y|W}$ for i=1,…,N.

**Collaborative/decentralized learning/inference**: In the learning phase, training data is used to infer the model parameter *W*, enabling the agents in the inference phase to make predictions about test label *Y* given the test feature vector X based on the model $P_{X,Y|W}$. Either or both learning and inference phases can be carried out collaboratively by the agents or in a decentralized fashion (i.e., separately by each agent). When collaborating for learning or inference, the *K* agents share their locally observed feature data via the aggregator. The operation of the aggregator is modelled as a stochastic aggregation mapping $P_{\hat{X}|X_{1},\ldots ,X_{k}}=P_{\hat{X}|X}$ from the input *K* local features to an output shared feature $\hat{X}$, to be used by each of the *K* local agents. As detailed next, for learning, the mapping $P_{\hat{X}|X}$ is applied independently to each data point. Furthermore, as we also detail later in this section, we impose privacy constraints on the aggregation mapping $P_{\hat{X}|X}$ so that the shared feature $\hat{X}$ does not reveal too much information about the local agents’ features.

We specifically distinguish the following four settings:

- *Collaborative learning–collaborative inference* (CL/CI): Agents collaborate during both learning and inference phases by sharing information about their respective features. Accordingly, during learning, each agent has access to the shared training data features $\hat{X}=\left({\hat{X}}_{1},\ldots ,{\hat{X}}_{N}\right)$, where each *n*th component ${\hat{X}}_{n}∼P_{\hat{X}|X=X_{n}}$ is generated independently by the aggregator in response to the observed feature vector $X_{n}$, in addition to its own observed local feature data $X_{k}$. Furthermore, during inference, agent *k* can use the shared test feature $\hat{X}∼P_{\hat{X}|X=X}$, obtained by aggregating the test feature vector X, in addition to its own observation $X_{k}$, in order to predict the test label *Y*.
- *Collaborative learning–decentralized inference* (CL/DI): Agents collaborate only during learning by sharing information about their respective features as explained above, while inference is decentralized. Accordingly, during inference, each *k*th agent uses the *k*th feature $X_{k}$ of test feature vector X in order to predict the test label *Y*.
- *Decentralized learning–collaborative inference* (DL/CI): Agents collaborate for inference, while each *k*th agent is allowed to use only its observed training data $X_{k}$, along with the labels Y shared by the aggregator, during learning.
- *Decentralized learning–decentralized inference* (DL/DI): Agents operate independently, with no cooperation in either learning or inference phases.

**Privacy constraints**: The aggregation mapping $P_{\hat{X}|X}$ shares the output feature $\hat{X}$ with each of the *K* local agents during collaborative learning and/or inference. To account for privacy constraints concerning agents’ data, we limit the amount of information that a “curious” eavesdropper may be able to obtain about the local features’ data from observing $\hat{X}$. To this end, we impose the following privacy constraint on the aggregation mapping so that the shared feature $\hat{X}$ does not leak too much information about the local features $X_{k}$ of all agents k=1,…,K.

The aggregation mapping $P_{\hat{X}|X}$ is said to be ϵ- individually private if

(2) $$\begin{array}{c} I(\hat{X};X_{k}|X^{\left(-k\right)})\leq \epsilon ,\;\mathrm{for}\;\mathrm{all}\;k=1,\ldots ,K, \end{array}$$

where $X^{\left(-k\right)}=\left(X_{1},\ldots ,X_{k-1},X_{k+1},\ldots ,X_{K}\right)$ and

$$I\left(\hat{X};X_{k}|X^{\left(-k\right)}\right)=E_{P_{\hat{X},X}}\left[\log\frac{P_{\hat{X},X_{k}|X^{\left(-k\right)}}}{P_{\hat{X}|X^{\left(-k\right)}}P_{X_{k}|X^{\left(-k\right)}}}\right]$$

is the conditional mutual information under the joint distribution $P_{\hat{X},X}=P_{X}P_{\hat{X}|X}$, with $P_{X}$ being the marginal of $P_{X,Y,W}$. The constraint (2) measures privacy against a strong eavesdropper that knows all features except the *k*th feature $X_{k}$. Specifically, the conditional mutual information $I(\hat{X};X_{k}|X^{\left(-k\right)})$ quantifies the additional information about $X_{k}$ gained by the eavesdropper upon observing the shared feature $\hat{X}$. As such, the metric is also relevant as a privacy measure against “curious” agents.

We note that although the privacy constraint in (2) bears a resemblance to the MI-differential privacy (MI-DP) constraint introduced in [9], the condition (2) does not have the same operational meaning. In fact, the MI-DP constraint in [9,10] or the *f*-divergence-based DP constraint in [11] ensure differential privacy for individual i.i.d. data samples of a training data set, and they rely on a mechanism that applies to the entire data set during learning. In contrast, the constraint (2) accounts for the privacy of correlated local features via a per-sample masking mechanism, and it applies to both learning and inference phases.

**Predictive loss under privacy constraints**: In all the four settings described above, any agent *k* uses the available training data $\left({\tilde{X}}_{k},Y\right)$, with ${\tilde{X}}_{k}$ being equal to $X_{k}$ for decentralized learning and to $\left(X_{k},\hat{X}\right)$ for collaborative learning, in order to infer the model parameter *W*. The inferred model is then used to predict the label *Y* given the test feature input ${\tilde{X}}_{k}$, with ${\tilde{X}}_{k}$ being equal to $X_{k}$ for decentralized inference and to $\left(X_{k},\hat{X}\right)$ for collaborative learning. We impose that the aggregation mapping $P_{\hat{X}|X}$ must satisfy the privacy constraint in (2).

The joint operation of learning and inference at agent *k* can be accordingly described via a stochastic predictive distribution $Q_{Y|{\tilde{X}}_{k},Y,{\tilde{X}}_{k}}$ on the test label *Y* given the training data $\left({\tilde{X}}_{k},Y\right)$ and test feature input ${\tilde{X}}_{k}$. The predictive distribution can be thought of as the result of a two-step application of learning and inference, where a model parameter is first learned using the input training data $\left({\tilde{X}}_{k},Y\right)$ and is subsequently used to infer the label corresponding to the test feature input ${\tilde{X}}_{k}$. Note that this stochastic mapping can account for arbitrary choices of learning and inference algorithms. By optimizing over aggregation mapping as well as over learning and inference algorithms, we define the ϵ-private predictive loss as

(3) $$\begin{array}{cc} R(\epsilon ) & =\min_{\begin{array}{c} P_{\hat{X}|X}\\ \in P(\hat{X}|X) \end{array}}\max_{k=1,\ldots ,K}\min_{\begin{array}{c} Q_{Y|{\tilde{X}}_{k},Y,{\tilde{X}}_{k}}\\ \in Q(Y|{\tilde{X}}_{k},Y,{\tilde{X}}_{k}) \end{array}}E_{P_{Y,{\tilde{X}}_{k},Y,{\tilde{X}}_{k}}}\left[-\log Q_{Y|{\tilde{X}}_{k},Y,{\tilde{X}}_{k}}\right]\\ \; & \;s.t\;I(\hat{X};X_{k}|X^{\left(-k\right)})\leq \epsilon \;\mathrm{for}\;\mathrm{all}\;k=1,\ldots ,K. \end{array}$$

In (3), the aggregation mapping $P_{\hat{X}|X}$ is optimized over some specified family $P(\hat{X}|X)$ of conditional distributions $P_{\hat{X}|X}$ in order to minimize the worst-case predictive loss across the agents under constraint (2). Furthermore, the inner optimization is over a class of predictive distributions $Q(Y|{\tilde{X}}_{k},Y,{\tilde{X}}_{k})$.

In the absence of privacy constraints (i.e., when ϵ=∞), assuming that the distribution family $P(\hat{X}|X)$ is sufficiently large, the optimal aggregation mapping $P_{\hat{X}|X}$ puts its entire mass on the output shared feature $\hat{X}=X$. As such, under collaborative learning, each agent *k* uses the entire feature data (i.e., ${\tilde{X}}_{k}=X$), and under collaborative inference, it uses the entire test feature vector ${\tilde{X}}_{k}=X$. The predictive loss (3) in the absence of privacy constraints is evaluated as

(4) $$\begin{array}{c} R\left(\infty \right)=\max_{k=1,\ldots ,K}\min_{\begin{array}{c} Q_{Y|{\tilde{X}}_{k},Y,{\tilde{X}}_{k}}\\ \in Q(Y|{\tilde{X}}_{k},Y,{\tilde{X}}_{k}) \end{array}}E_{P_{Y,{\tilde{X}}_{k},Y,{\tilde{X}}_{k}}}\left[-\log Q_{Y|{\tilde{X}}_{k},Y,{\tilde{X}}_{k}}\right]. \end{array}$$

The predictive loss (4) represents the worst-case minimum average cross-entropy loss across all agents, which can be obtained given the information about the training data set and the test input feature [5].

## 3. Preliminaries and Fully Collaborative Benchmark

In this section, we first provide a brief explanation of the main information-theoretic metrics used in this work. Then, we define and derive the average predictive loss for the benchmark case in which both learning and inference are collaborative.

**Information-theoretic metrics**: Let *A* and *B* denote two (discrete or continuous) random variables with joint distribution $P_{A,B}$, and with corresponding marginals $P_{A}$ and $P_{B}$. The joint entropy of *A* and *B*, denoted H(A,B), is defined as $H\left(A,B\right)=E_{P_{A,B}}\left[-\log P_{A,B}\right]$, with $E_{P}\left[\cdot \right]$ denoting the expectation with respect to distribution *P*. More generally, the conditional entropy of *A* given *B* is defined as $H\left(A|B\right)=E_{P_{A,B}}\left[-\log P_{A|B}\right]$, where $P_{A|B}=P_{A,B}/P_{B}$ is the conditional distribution of *A* given *B*. By the chain rule, we have the relationship H(A,B)=H(B)+H(A|B); we also have the property that conditioning does not increase entropy [12] (i.e., H(A|B)≤H(A)). The mutual information I(A;B) between the random variables is defined as $I\left(A;B\right)=E_{P_{A,B}}\left[\log\left(\frac{P_{A,B}}{P_{A}P_{B}}\right)\right].$ Finally, for random variables A,B, and *C* with joint distribution $P_{A,B,C}$, the conditional mutual information I(A;B|C) between *A* and *B* given *C* is defined as $I\left(A;B|C\right)=E_{P_{A,B,C}}\left[\log\left(\frac{P_{A,B|C}}{P_{A|C}P_{B|C}}\right)\right]$.

**Private collaborative learning–collaborative inference** (CL/CI): As a benchmark, we now study the predictive loss (3) for the CL/CI setting. The ϵ-private predictive loss (3) of CL/CI is given as

(5) $$R^{\mathrm{CL}/\mathrm{CI}}\left(\epsilon \right)=\min_{P_{\hat{X}|X}\in F\left(\hat{X}|X\right)}\max_{k=1,\ldots ,K}\min_{\begin{array}{c} Q_{Y|\hat{X},X_{k},Y,\hat{X},X_{k}}\\ \in Q(Y|\hat{X},X_{k},Y,\hat{X},X_{k}) \end{array}}E_{P_{Y,\hat{X},X_{k},Y,\hat{X},X_{k}}}\left[-\log Q_{Y|\hat{X},X_{k},Y,\hat{X},X_{k}}\right]$$

where

(6) $$\begin{array}{c} F\left(\hat{X}|X\right)=\left\{P_{\hat{X}|X}\in P\left(\hat{X}|X\right):\mathrm{constraint}\;(2)\;\mathrm{holds}\right\} \end{array}$$

is the feasible space of conditional distributions satisfying the privacy constraint (2). The following lemma presents an information-theoretic characterization of the loss $R^{\mathrm{CL}/\mathrm{CI}}\left(\epsilon \right)$.

**Lemma** **1.** *Assume that the family $Q(Y|\hat{X},X_{k},Y,\hat{X},X_{k})$ comprises the set of all predictive distributions $Q_{Y|\hat{X},X_{k},Y,\hat{X},X_{k}}$. Then, the ϵ-private predictive loss (5) for the CL/CI setting evaluates as*

(7) $$\begin{array}{c} R^{\mathrm{CL}/\mathrm{CI}}\left(\epsilon \right)=\min_{\begin{array}{c} P_{\hat{X}|X}\\ \in F(\hat{X}|X) \end{array}}\max_{k=1,\ldots ,K}H\left(Y|\hat{X},X_{k},Y,\hat{X},X_{k}\right). \end{array}$$

*In addition, if ϵ=∞, and $P(\hat{X}|X)$ includes the space of all conditional distributions $P_{\hat{X}|X}$, then the predictive loss (4) in the absence of privacy constraints for CL/CI is evaluated as*

(8) $$\begin{array}{c} R^{\mathrm{CL}/\mathrm{CI}}\left(\infty \right)=H\left(Y|X,X,Y\right). \end{array}$$

**Proof.**  For a fixed aggregation mapping $P_{\hat{X}|X}$, and an agent *k*, the predictive distribution that minimizes the inner cross entropy term in (5), $E_{P_{Y,\hat{X},X_{k},Y,\hat{X},X_{k}}}\;\left[\;-\log Q_{Y|\hat{X},X_{k},Y,\hat{X},X_{k}}\right]$, is the posterior distribution, $P_{Y|\hat{X},X_{k},Y,\hat{X},X_{k}}$ [12], resulting in the conditional entropy term in (7). When ϵ=∞ and $P(\hat{X}|X)$ includes the space of all conditional distributions, we have $\hat{X}=X$ and $\hat{X}=X$, yielding (8). □

## 4. Cost of Decentralization under Privacy Constraints

In this section, we use the benchmark predictive loss (7) observed under the ideal CL/CI setting to evaluate the cost of decentralization in the learning and/or inference phases under privacy constraints.

**Lemma** **2.** *The ϵ-private predictive losses of decentralized learning and/or inference are given as*

(9) $$\begin{array}{cc} R^{\mathrm{CL}/\mathrm{DI}}\left(\epsilon \right) & =\min_{P_{\hat{X}|X}\in F\left(\hat{X}|X\right)}\max_{k=1,\ldots ,K}H\left(Y|X_{k},X_{k},\hat{X},Y\right) \end{array}$$

(10) $$\begin{array}{cc} R^{\mathrm{DL}/\mathrm{CI}}\left(\epsilon \right) & =\min_{P_{\hat{X}|X}\in F\left(\hat{X}|X\right)}\max_{k=1,\ldots ,K}H\left(Y|X_{k},\hat{X},X_{k},Y\right) \end{array}$$

(11) $$\begin{array}{cc} R^{\mathrm{DL}/\mathrm{DI}}\left(\epsilon \right) & =\max_{k=1,\ldots ,K}H\left(Y|X_{k},X_{k},Y\right), \end{array}$$

*where set $F(\hat{X}|X)$ is as defined in (6).*

**Proof.**  The result is a direct extension of Lemma 1 to CL/DI, DL/CI, and DL/DI. □

Note that the predictive loss (11) of the fully decentralized DL/DI setting does not depend on the privacy parameter ϵ, since decentralization does not entail any privacy loss. Therefore, in the absence of privacy constraints, we have $R^{\mathrm{DL}/\mathrm{DI}}\left(\infty \right)=R^{\mathrm{DL}/\mathrm{DI}}\left(\epsilon \right)$, while the predictive losses in (9) and (10) evaluate as

(12) $$\begin{array}{cc} R^{\mathrm{CL}/\mathrm{DI}}\left(\infty \right) & =\max_{k=1,\ldots ,K}H\left(Y|X_{k},X,Y\right), \end{array}$$

(13) $$\begin{array}{cc} R^{\mathrm{DL}/\mathrm{CI}}\left(\infty \right) & =\max_{k=1,\ldots ,K}H\left(Y|X,X_{k},Y\right), \end{array}$$

under the assumption of sufficiently large $P(\hat{X}|X)$. Furthermore, using the property that conditioning does not increase entropy [8] results in the following relation between the predictive losses of the four schemes—CL/CI, CL/DI, DL/CI and DL/DI—in the absence of privacy constraints:

(14) $$\begin{array}{cc} R^{\mathrm{CL}/\mathrm{CI}}\left(\infty \right) & \leq \min\{R^{\mathrm{CL}/\mathrm{DI}}\left(\infty \right),R^{\mathrm{DL}/\mathrm{CI}}\left(\infty \right)\}\\ & \leq \max\{R^{\mathrm{CL}/\mathrm{DI}}\left(\infty \right),R^{\mathrm{DL}/\mathrm{CI}}\left(\infty \right)\}\\ & \leq R^{\mathrm{DL}/\mathrm{DI}}\left(\infty \right). \end{array}$$

The difference between the ϵ-private predictive risks of the decentralized and collaborative schemes captures the *cost of decentralization*. Specifically, given two schemes a,b∈{CL/CI, CL/DI, DL/CI, DL/DI} such that $R^{a}\left(\epsilon \right)\geq R^{b}\left(\epsilon \right)$, we define the cost of *a* with respect to *b* as

(15) $$\begin{array}{c} C^{a-b}\left(\epsilon \right)=R^{a}\left(\epsilon \right)-R^{b}\left(\epsilon \right). \end{array}$$

In the absence of privacy constraints (ϵ=∞) and assuming symmetric agents so that the maximum in (4) is attained for any k=1,…,K, the cost of decentralization can be exactly characterized as in the following result.

**Proposition** **1.** *The cost of decentralization (15) for ϵ=∞ and symmetric agents can be characterized for the kth learning agent as detailed in Table 1, where $X^{\left(-k\right)}=\left(X_{1},\ldots ,X_{k-1},X_{k+1},\ldots ,X_{K}\right)$ and $X^{\left(-k\right)}=\left(X_{1},\ldots ,X_{k-1},X_{k+1},\ldots ,X_{K}\right)$.*

**Proof.**  We illustrate the derivation of the cost of decentralization between CL/DI and CL/CI, as the proof can be similarly completed. In the absence of privacy constraints and assuming symmetric agents, we have from (8) and (12), $C^{\mathrm{CL}/\mathrm{DI}-\mathrm{CL}/\mathrm{CI}}\left(\infty \right)=H\left(Y|X_{k},X,Y\right)-H\left(Y|X,X,Y\right)=I\left(Y;X^{\left(-k\right)}|X_{k},X,Y\right).$ □

The results in Table 1 have intuitive interpretations. For instance, the cost $C^{\mathrm{CL}/\mathrm{DI}-\mathrm{CL}/\mathrm{CI}}\left(\infty \right)$$=I(Y;X^{\left(-k\right)}|X_{k},X,Y)$ corresponds to the additional information about label *Y* that can be obtained from observing the features $X^{\left(-k\right)}$ of other agents, given X,Y, and $X_{k}$. Examples will be provided in the next section in which the cost of decentralization is evaluated also in the presence of privacy constraints based on (7), (9)–(11).

## 5. Examples and Remarks

In this section, we consider two simple numerical examples to illustrate the cost of decentralization for learning and/or inference with and without the privacy constraints that were quantified in Section 4 for general models. We note that evaluating the derived metrics for real-world examples would generally require the implementation of mutual information estimators, and is left for future work.

### 5.1. Two-Agent Non-Private Collaborative Learning (CL) and/or Inference (CI)

Consider two agents (K=2) observing binary joint features $X_{1},X_{2}\in \left\{0,1\right\}$, which have the joint distribution defined by the probability *r* of the two features $X_{1}$ and $X_{2}$ being equal, that is, $\mathrm{Pr}\left[X_{1}=X_{2}|X_{2}=x_{2}\right]=r/2,\;with\;\mathrm{Pr}\left[X_{1}=1\right]=\mathrm{Pr}\left[X_{2}=1\right]=0.5.$ Parameter *r* quantifies the statistical dependencies between features $X_{1}$ and $X_{2}$ through the MI $I\left(X_{1};X_{2}\right)=\log2-H_{b}\left(r\right)$, where $H_{b}\left(r\right)=-r\log\left(r\right)-\left(1-r\right)\log\left(1-r\right)$ denotes the binary entropy with parameter *r*. Note that the MI takes the maximum value of $I(X_{1};X_{2})=1$ when r=0 or 1, and the minimum value of $I(X_{1};X_{2})=0$ when r=0.5. The output binary label Y∈{0,1} depends on the feature vector X through the model

(16) $$\begin{array}{c} P_{Y=1|X_{1},X_{2},W}=\left\{\begin{array}{cc} W_{1} & \mathrm{if}\;X_{1}⊕X_{2}=0\\ W_{2} & \mathrm{if}\;X_{1}⊕X_{2}=1 \end{array}\right., \end{array}$$

with model parameters $W=(W_{1},W_{2})$, where $\left\{W_{1},W_{2}\right\}\in \left[0,1\right]$. Accordingly, $W_{1}$ and $W_{2}$ are the probabilities of the event Y=1 when $X_{1}$ and $X_{2}$ are equal or different, respectively. We assume that the model parameters are a priori independent and distributed according to beta distributions ([8], Section 2.4.2) as $P_{W_{1},W_{2}}=\mathrm{Beta}\left(W_{1}|\alpha_{1},\beta_{1}\right)\mathrm{Beta}\left(W_{2}|\alpha_{2},\beta_{2}\right),$ where $\alpha_{1},\beta_{1},\alpha_{2},\beta_{2}>0$ are fixed hyperparameters.

**Figure 2:**
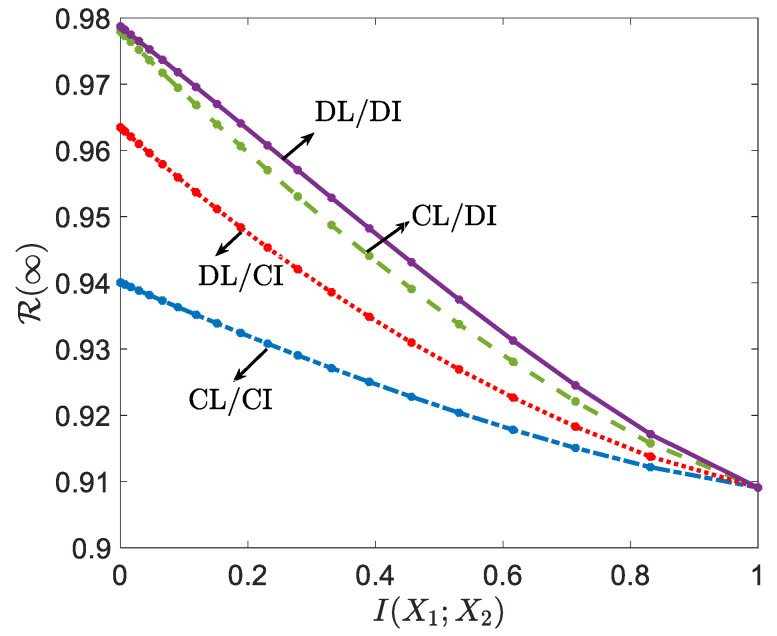
Predictive losses (7), (9)–(11) for the four schemes under no privacy constraints (ϵ=∞) as a function of the mutual information $I(X_{1};X_{2})$. ($\alpha_{1}=2$, $\beta_{1}=1.5$, $\alpha_{2}=1.5$, $\beta_{2}=2$, and N=3).

Figure 2 compares the predictive loss derived in Lemma 2 with no privacy constraints (ϵ=∞) under the four schemes—CL/CI, CL/DI, DL/CI and DL/DI—as a function of the mutual information $I(X_{1};X_{2})$ between the components of the bivariate feature vector. The number of data samples is N=3, and other hyperparameters are set to $\alpha_{1}=2$, $\beta_{1}=1.5$, $\alpha_{2}=1.5$, and $\beta_{2}=2$. When the MI $I(X_{1};X_{2})$ is large, the predictive risks under collaborative and decentralized schemes are similar, and the cost of decentralization is negligible. This is because a larger MI $I(X_{1};X_{2})$ implies that each local agent’s feature $X_{k}$, for k=1,2, is highly informative about the local feature $X^{\left(-k\right)}$ of the other agent, and no significant additional information can be obtained via collaboration. This applies to both learning and inference phases. Conversely, when the MI is small, decentralization entails a significant cost. In this example, centralized inference is more effective than centralized learning due to the importance of having access to both $X_{1}$ and $X_{2}$ in order to infer *Y* by (16).

### 5.2. Three-Agent Private CL and/or CI

We now extend the example in Section 5.1 by considering three agents (K=3) and by imposing privacy constraints during collaboration in the learning and inference phases. The feature vector $X=(X_{1},X_{2},X_{3})$ consists of three binary features $X_{k}\in \left\{0,1\right\}$ for k=1,2,3, where $X_{1}$ and $X_{2}$ are distributed as in Section 5.1, and we have $\mathrm{Pr}\left[X_{3}|X_{1}=x_{1},X_{2}=x_{2}\right]=\mathrm{Pr}\left[X_{3}|X_{2}=x_{2}\right]$ with $\mathrm{Pr}[X_{3}\neq X_{2}|X_{2}=x_{2}]=1-r$. Generalizing the previous example, the output binary label Y∈{0,1} depends on the feature vector X through the model

(17) $$\begin{array}{c} P_{Y=1|X,W}=\left\{\begin{array}{cc} W_{1} & \mathrm{if}\;X_{1}⊕X_{2}⊕X_{3}=0\\ W_{2} & \mathrm{if}\;X_{1}⊕X_{2}⊕X_{3}=1 \end{array}\right., \end{array}$$

where model parameters have the same prior distribution. The aggregation mapping $P_{\hat{X}|X}$ produces a binary random variable $\hat{X}\in \left\{0,1\right\}$ as $\hat{X}=X_{1}⊕X_{2}⊕X_{3}⊕\xi ,$ with ξ∼Bern(s), where the noise variable ξ∼Bern(s) is chosen independently of the feature vector X, and the parameter s∈[0,1] is selected so as to guarantee the privacy constraints in (2), which can be written as

$$\begin{array}{cc} \epsilon \geq & \max\{-H_{b}\left(s\right)+H_{b}\left(s\left(1-r\right)+r\left(1-s\right)\right),-H_{b}\left(s\right)+\\ & H_{b}\left(sr+\left(1-r\right)\left(1-s\right)\right),-H_{b}\left(s\right)+2r\left(1-r\right)\log\left(2\right)\\ & +\left({\left(1-r\right)}^{2}+r^{2}\right)H_{b}\left(\frac{{\left(1-r\right)}^{2}s+r^{2}\left(1-s\right)}{{\left(1-r\right)}^{2}+r^{2}}\right)\}. \end{array}$$

**Figure 3:**
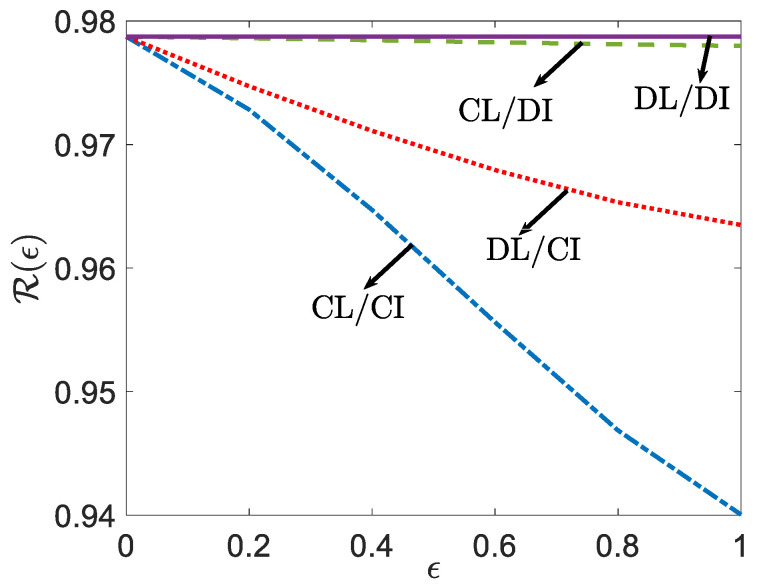
Predictive losses (7), (9)–(11) for the four schemes as a function of privacy measure ϵ. ($\alpha_{1}=2$, $\beta_{1}=1.5$, $\alpha_{2}=1.5$, $\beta_{2}=2$ and N=3).

Figure 3 compares the predictive loss R(ϵ) derived in Lemma 2 of the four schemes—CL/CI, CL/DI, DL/CI and DL/DI—as a function of the privacy parameter ϵ for fixed r=0.5. In the high-privacy regime, where ϵ is small, the shared feature $\hat{X}$ is not informative about the local observed features, and collaborative learning/inference brings little benefit over the decentralized schemes. However, as ϵ increases, thereby weakening privacy requirements, the shared feature $\hat{X}$ becomes more informative about the observed feature vector X, and the cost of decentralization becomes increasingly significant, reaching its maximum value under no privacy (i.e., when ϵ=1).

The examples studied in this section are simple enough to exactly evaluate the MI terms, but sufficiently rich to clearly demonstrate the cost of decentralization arising in the four collaboration settings of CL/CI, CL/DI, DL/CI, and DL/DI. They elucidate a simple vertical FL setting with features partitioned across agents and a discriminative model as given in (16).

## 6. Conclusions

This paper presents a novel information-theoretic characterization of the cost of decentralization during learning and/or inference in a vertical FL setting. Under privacy constraints on the aggregation mechanism that enables inter-agent communications, we show, by adopting a Bayesian framework, that the average predictive performance of the four schemes can be quantified in terms of conditional entropy terms. Furthermore, when no privacy constraints are imposed, the cost of decentralization for symmetric agents is shown to be exactly characterized by conditional mutual information terms.

The proposed information-theoretic framework is relevant for real-world vertical FL settings, such as credit scoring in banking [13], healthcare [14], and smart retailing. We leave the investigation of practical implications of the analysis via efficient MI estimators, such as the mutual information neural estimators (MINE) [15], to future research.

## Figures and Tables

**Table 1:** Cost of decentralization $C^{a-b}\left(\infty \right)$ (*a* defines the column and *b* the row).

	CL/CI	CL/DI	DL/CI	DL/DI
CL/CI	–	$I(Y;X^{\left(-k\right)}|X_{k},X,Y)$	$I(Y;X^{\left(-k\right)}|X,X_{k},Y)$	$I(Y;X^{\left(-k\right)},X^{\left(-k\right)}|X_{k},X_{k},Y)$
CL/DI	–	–		$I(Y;X^{\left(-k\right)}|X_{k},X_{k},Y)$
DL/CI	–		–	$I(Y;X^{\left(-k\right)}|X_{k},X_{k},Y)$
DL/DI	–	–	–	–
